# Acclimation of intertidal macroalgae *Ulva prolifera* to UVB radiation: the important role of alternative oxidase

**DOI:** 10.1186/s12870-024-04762-w

**Published:** 2024-02-28

**Authors:** Jinhui Xu, Xinyu Zhao, Yi Zhong, Tongfei Qu, Baixue Sun, Huanxin Zhang, Chengzong Hou, Zhipeng Zhang, Xuexi Tang, Ying Wang

**Affiliations:** 1 College of Marine Life Sciences, Ocean University of China, 5 Yushan Road, Qingdao, 266003 China; 2 Laboratory for Marine Ecology and Environmental Science, Qingdao Marine Science and Technology Center, 1 Wenhai Road, Qingdao, 266237 China; 3 Laoshan Laboratory, 1 Wenhai Road, Qingdao, 266237 China; 4 College of Geography and Environment, Shandong Normal University, 1 Daxue Road, Jinan, 250000 China; 5 Tianjin Research Institute for Water Transport Engineering, Ministry of Transport, Tianjin, 300456 China

**Keywords:** Alternative oxidase, Photosynthesis optimization, ROS homeostasis, Photosynthetically active radiation, Ultraviolet-B, *Ulva prolifera*

## Abstract

**Background:**

Solar radiation is primarily composed of ultraviolet radiation (UVR, 200 − 400 nm) and photosynthetically active radiation (PAR, 400 − 700 nm). Ultraviolet-B (UVB) radiation accounts for only a small proportion of sunlight, and it is the primary cause of plant photodamage. The use of chlorofluorocarbons (CFCs) as refrigerants caused serious ozone depletion in the 1980s, and this had led to an increase in UVB. Although CFC emissions have significantly decreased in recent years, UVB radiation still remains at a high intensity. UVB radiation increase is an important factor that influences plant physiological processes. *Ulva prolifera*, a type of macroalga found in the intertidal zone, is intermittently exposed to UVB. Alternative oxidase (AOX) plays an important role in plants under stresses. This research examines the changes in AOX activity and the relationships among AOX, photosynthesis, and reactive oxygen species (ROS) homeostasis in *U. prolifera* under changes in UVB and photosynthetically active radiation (PAR).

**Results:**

UVB was the main component of solar radiation impacting the typical intertidal green macroalgae *U. prolifera*. AOX was found to be important during the process of photosynthesis optimization of *U. prolifera* due to a synergistic effect with non-photochemical quenching (NPQ) under UVB radiation. AOX and glycolate oxidase (GO) worked together to achieve NADPH homeostasis to achieve photosynthesis optimization under changes in PAR + UVB. The synergism of AOX with superoxide dismutase (SOD) and catalase (CAT) was important during the process of ROS homeostasis under PAR + UVB.

**Conclusions:**

AOX plays an important role in the process of photosynthesis optimization and ROS homeostasis in *U. prolifera* under UVB radiation. This study provides further insights into the response of intertidal macroalgae to solar light changes.

**Supplementary Information:**

The online version contains supplementary material available at 10.1186/s12870-024-04762-w.

## Background

Solar radiation is composed of ultraviolet radiation (UVR, 200 − 400 nm), photosynthetically active radiation (PAR, 400 − 700 nm), and infrared wavelengths (760 − 1000 nm). UVR includes ultraviolet-A (UVA, 320–400 nm), ultraviolet-B (UVB, 280 − 320nm), and ultraviolet-C (UVC, 200–280 nm). The UVC wavelength is short and largely absorbed by ozone, preventing it from reaching the Earth's surface. UVA has the longest wavelength and weakest energy, and can directly reach the Earth's surface. UVA, which can cause both inhibitory and enhancing effects on biomass morphology and photosynthesis in plants, is less efficient than UVB in mediating some biological responses [1]. UVB radiation accounts for only a small proportion of sunlight, while it is easily absorbed by biological macromolecules such as proteins, lipids, and nucleic acids [2]. In addition, it affects the growth and reproduction of organisms, physiology, and the redox state, and causes DNA damage. This damage includes decreasing the growth rate, increasing the death rate of thalli [3], delaying spore germination [4], affecting the activities of photosystem II (PSII) and Rubisco, reducing the levels of chlorophyll and carotenoids, destroying the ultrastructure of chloroplasts [5], producing excessive reactive oxygen species [4], and forming cyclobutane pyrimidine dimer (CPD), even leading to the death of plant mutants lacking specific DNA repair pathways [6]. The use of chlorofluorocarbons (CFCs) as refrigerants caused a serious depletion of ozone in the 1980s, and this led to an increase in UVB radiation that might induce photodamage in plants. Although CFC emissions have decreased significantly in recent years, UVB radiation still has remained at a high intensity. PAR is the energy source for plants and organic matter synthesis, and excess PAR may lead to different effects on plants [7]. Under natural conditions, a high level of UVB radiation occurs simultaneously with high PAR irradiance [8]. High levels of PAR can cause an accumulation pattern in barley with distinct tolerance to UVR, which is associated with the negative effects of UVB on photosynthesis [9]. It has been reported that both UVB and PAR can induce photoprotective responses in plants [8], with UVB being often a major factor inducing photodamage [10, 11]. The intertidal zone is the transitional zone between the land and the sea. Macroalgae living in the intertidal zone are faced with intermittent solar radiation due to tidal changes. *Ulva prolifera* is a type of typical intertidal green macroalgae [12–14]. Macroalgae have mechanisms to acclimate to changes in UVB radiation, including the accumulation of flavonoids and antioxidants [8]. Furthermore, the responses of non-photochemical quenching (NPQ) and antioxidant enzymes play important roles in the ability of thalli to acclimate to the environment under UVB and PAR radiation. NPQ plays important roles in acclimating to the environment under UVB radiation in macroalgae [15–17]. UVB radiation significantly increased NPQ, indicating that NPQ is an effective photoprotection strategy in green macroalgae *U. prolifera* [18], and similar results were also observed in red macroalgae *Neoporphyra haitanensis* [16, 18]. Furthermore, the induction of NPQ in *N. haitanensis* was dependent on delta pH [16]. The antioxidant system is also important during reactive oxygen species (ROS) homeostasis in macroalgae under UVB radiation, and there are significant differences in antioxidant system strategies under UVB conditions among various macroalgae [18]. In *N. haitanensis*, increased antioxidant enzyme activities and the accumulation of non-enzymatic antioxidants showed the positive response of enzymatic antioxidants to maintain the balance of ROS under low UVB conditions [19]. The activity of peroxidase (POX) in *Grateloupia filicina* increased significantly after exposure to UVB [20]. In contrast, catalase (CAT) in green macroalgae *U. rigida* rapidly increased its activity in response to radiation stress [10]. *Desmarestia anceps*, a brown macroalgae collected from the upper and mid-subtidal areas of Port Cove, King George Island, Antarctica, exhibited high superoxide dismutase (SOD) activity, which was also supported by considerable glutathione reductase activities, enabling its tolerance to high UVB radiation [21].

Alternative oxidase (AOX) exists in plants in addition to the cytochrome oxidase (COX) respiratory pathway. The electrons that accept reductive ubiquinone directly reduce oxygen to water [22–25], so the electron flow of the AOX respiratory pathway bypasses the last two energy conservation sites (complexes III and IV) related to the COX respiratory pathway [26]. Because of this property, which has nothing to do with energy conservation, this can alleviate an imbalance in carbon and energy metabolism caused by stress [27]. Environmental factors can significantly affect the expression of AOX protein and AOX activity in plants [26, 28]. AOX is considered to play an important role in the ROS balance in cells and plants by maintaining the function of mitochondria [28–30]. AOX in peas [26], rice [31], and *Haematococcus pluvialis* [32] can consume an excessive reduction equivalent in chloroplasts, and it avoids excessive reduction in the photosynthetic electron transport chain. This can indirectly inhibit ROS production by photosynthesis, which can prevent oxidative damage of the thylakoid membrane and balance the effect of stress on photosynthesis. In addition, AOX showed the ability to optimize photosynthesis in plants under high light stress. High light stress can lead to a decrease in the photosynthetic rate of plants, and alternative oxidase plays a role in plant adaptation to this type of stress, including high levels of UVB radiation [33]. *AOX1a* knockout *Arabidopsis thaliana* exhibited low photosynthetic performance under high light conditions despite the presence of the malate-OAA shuttle and photorespiration, which can optimize plant photosynthesis [34]. AOX contributed to PSII photoprotection in C3 plants by maintaining photorespiration to detoxify glycolate via the indirect export of excess reducing equivalents from chloroplasts by the Mal/OAA shuttle. However, AOX did not respond to high light conditions and contributed little to PSII photoprotection in C4 leaves with little photorespiration possessing a highly active Mal/OAA shuttle [35]. In *Chlamydomonas reinhardtii*, a chloroplast-mitochondria coupling allowed for the dissipation of photosynthetically derived electrons via ROS reduction through AOX when photosynthetic electron carriers are highly reduced under high light [36]. There is relatively little research on the role of AOX during the environmental response of macroalgae. RNA-seq analysis demonstrated that AOX played an important role in stress resistance and invasion processes in the macroalgae *Caulerpa cylindracea* [37]. In *Auxenochlorella protothecoides*, AOX was essential in promoting ROS scavenging and maintaining redox homeostasis for algal chloroplast development during greening [30]. Previous studies have confirmed the significant roles of NPQ and the antioxidant system during defense against environmental stress in the common intertidal macroalgae *U. prolifera*. Furthermore, existing research has revealed a correlation between NPQ and antioxidant system during the stress response of *U. prolifera* [18, 38, 39]. Due to the crucial role of AOX in the plant stress response and the wide occurrence of UVB in the natural environment, it is necessary to explore the relationship between antioxidant systems, photoprotection, and ROS homeostasis in *U. prolifera* under UVB radiation.

In the present research, the physiological response strategies of the typical intertidal green macroalgae *U. prolifera* under changes in PAR and UVB were studied. This work focused on changes in AOX activity and the relationships among AOX, photosynthesis, and ROS homeostasis in *U. prolifera* under changes in UVB and PAR. This research provides a theoretical basis for a better understanding of the acclimation of macroalgae to adversity and a basis for accurately assessing the environmental impact of solar radiation.

## Results

### Detection of AOX

**Fig. 1:**
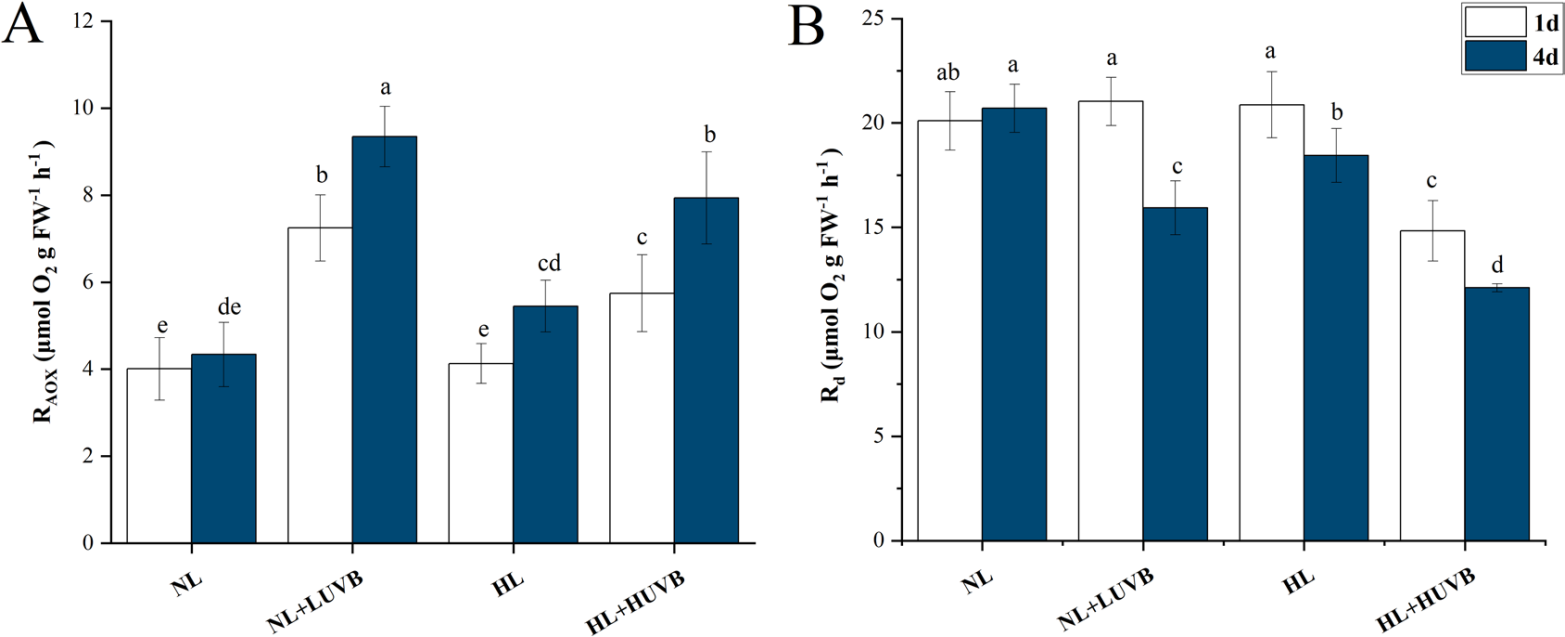
Effects of ultraviolet-B (UVB) and photosynthetically active radiation (PAR) on (**A**) AOX respiration rate (R_AOX_) and (**B**) the dark respiration rate (R_d_) in *Ulva prolifera* on the 1st day and the 4th day. All data are the mean values (± SD) from three biological replicates. Groups with different lowercase letters are significantly different (*P* < 0.05)

As shown in Fig. 1-A, the AOX respiration rate (R_AOX_) under NL + LUVB and HL + HUVB radiation increased significantly compared with the NL (*P* < 0.05). As shown in Fig. 1-B, the dark respiration rate (Rd) decreased significantly under NL + LUVB, HL, and HL + HUVB radiation on the 4th day, while it decreased significantly on the 1st day under HL + HUVB radiation (*P* < 0.05).

### Photosynthetic activity

**Fig. 2:**
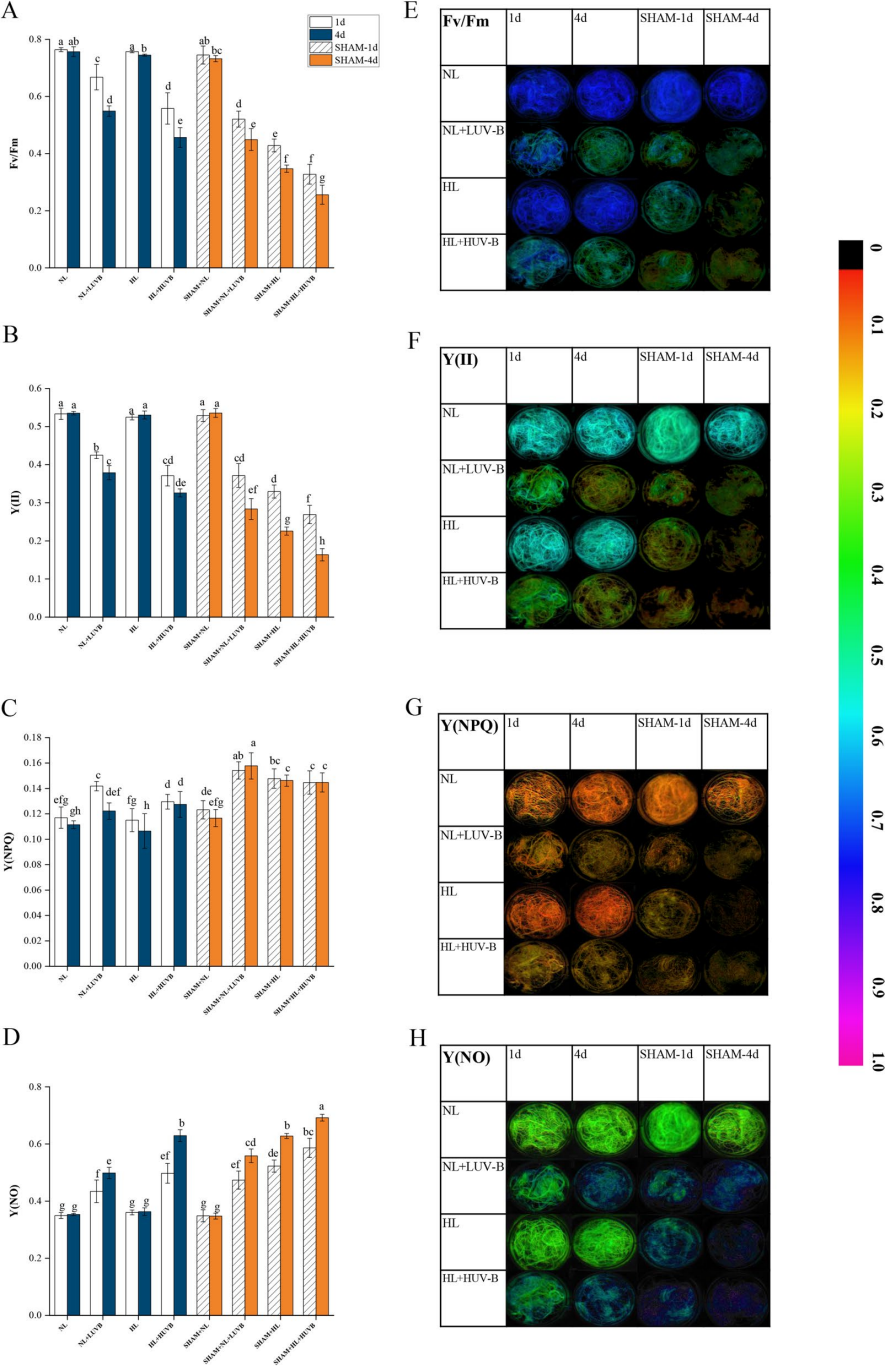
Effects of ultraviolet-B (UVB) and photosynthetically active radiation (PAR) on photosystem II (PS II) fluorescence parameters in *Ulva prolifera* on the 1st day and the 4th day. SHAM-1d and SHAM-4d show the changes of the SHAM group on the 1st day and the 4.^th^ day, respectively. **A** The maximum PSII quantum yield (*Fv/Fm*); **B** the effective PSII quantum yield (Y(II)); **C** the regulated non-photochemical quantum yield (Y(NPQ)); **D** the non-regulated non-photochemical quantum yield (Y(NO)); **E** images of the maximum PSII quantum yield (*Fv/Fm*); **F** images of the effective PSII quantum yield (Y(II)); **G** images of the regulated non-photochemical quantum yield (Y(NPQ)); **H** images of the non-regulated non-photochemical quantum yield (Y(NO)). The bar chart in the picture shows the relative percentage values of Fv/ Fm, Y(II), Y(NPQ), and Y(NO). All data are the mean values (± SD) from three biological replicates. Groups with different lowercase letters are significantly different (*P* < 0.05)

**Fig. 3:**
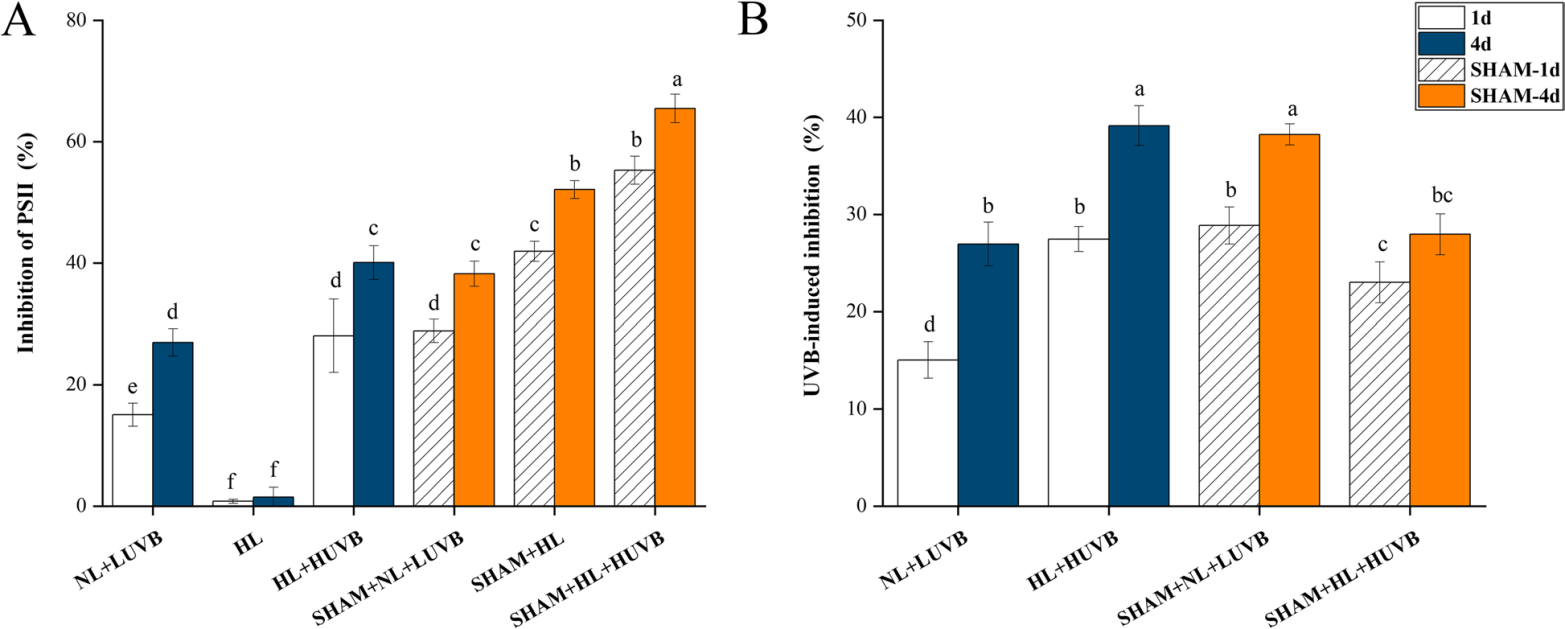
Effects of ultraviolet-B (UVB) and photosynthetically active radiation (PAR) on photosystem II (PS II) inhibition parameters in *Ulva prolifera* on the 1st day and the 4th day. SHAM-1d and SHAM-4d show the changes of the SHAM group on the 1st day and the 4.^th^ day, respectively. **A** The inhibition of PS II due to high PAR or PAR + UVB; **B** extra UVB-induced inhibition compared with PAR alone. All data are the mean values (± SD) from three biological replicates. Groups with different lowercase letters are significantly different (*P* < 0.05)

As shown in Fig. 2-A, the maximum PSII quantum yield (*Fv/Fm*) value decreased significantly under NL + LUVB radiation (from 0.76 ± 0.01 to 0.67 ± 0.04). The *Fv/Fm* decreased significantly under HL + HUVB radiation compared with the thalli under NL + LUVB radiation on the 1st day (from 0.76 ± 0.01 to 0.56 ± 0.05) (*P* < 0.05). The decreasing trend increased significantly with the presence of salicylhydroxamic acid (SHAM). The trend of *Fv/Fm* on the 4th day was the same as that on day 1, while the *Fv/Fm* value on the 4th day was lower than that on day 1. The effective PSII quantum yield (Y(II)) showed a similar trend compared with the result of *Fv/Fm* (Fig. 2-B). As illustrated in Fig. 3, UVB inhibited the activity of PS II more significantly than PAR. The regulated non-photochemical quantum yield (Y(NPQ)) values increased significantly under UVB radiation compared with that under NL, and an increasing trend of Y(NPQ) was observed in the presence of SHAM. And the results of NPQ increased further in the presence of SHAM (Fig. 2-C). As shown in Fig. 2-D, the non-regulated non-photochemical quantum yield (Y(NO)) values increased significantly under UVB radiation, which further increased with the presence of SHAM. Figure 2-E to Fig. 2-H show the images measured with Imaging-PAM. Value of NPQ increased significantly under UVB radiation, which was the highest under NL + LUVB on day 1 without SHAM (Supplementary Figure S1). NPQ increased significantly under UVB radiation, with the highest mean value measured under NL + LUVB (day 1, without SHAM; Supplementary Figure S1). Upon addition of SHAM, NPQ further increased (*P* < 0.05), with the highest mean values measured in the condition SHAM + NL + LUVB (*P* < 0.05; Supplementary Figure S1).

### Changes in the rapid light curves (RLCs)

**Fig. 4:**
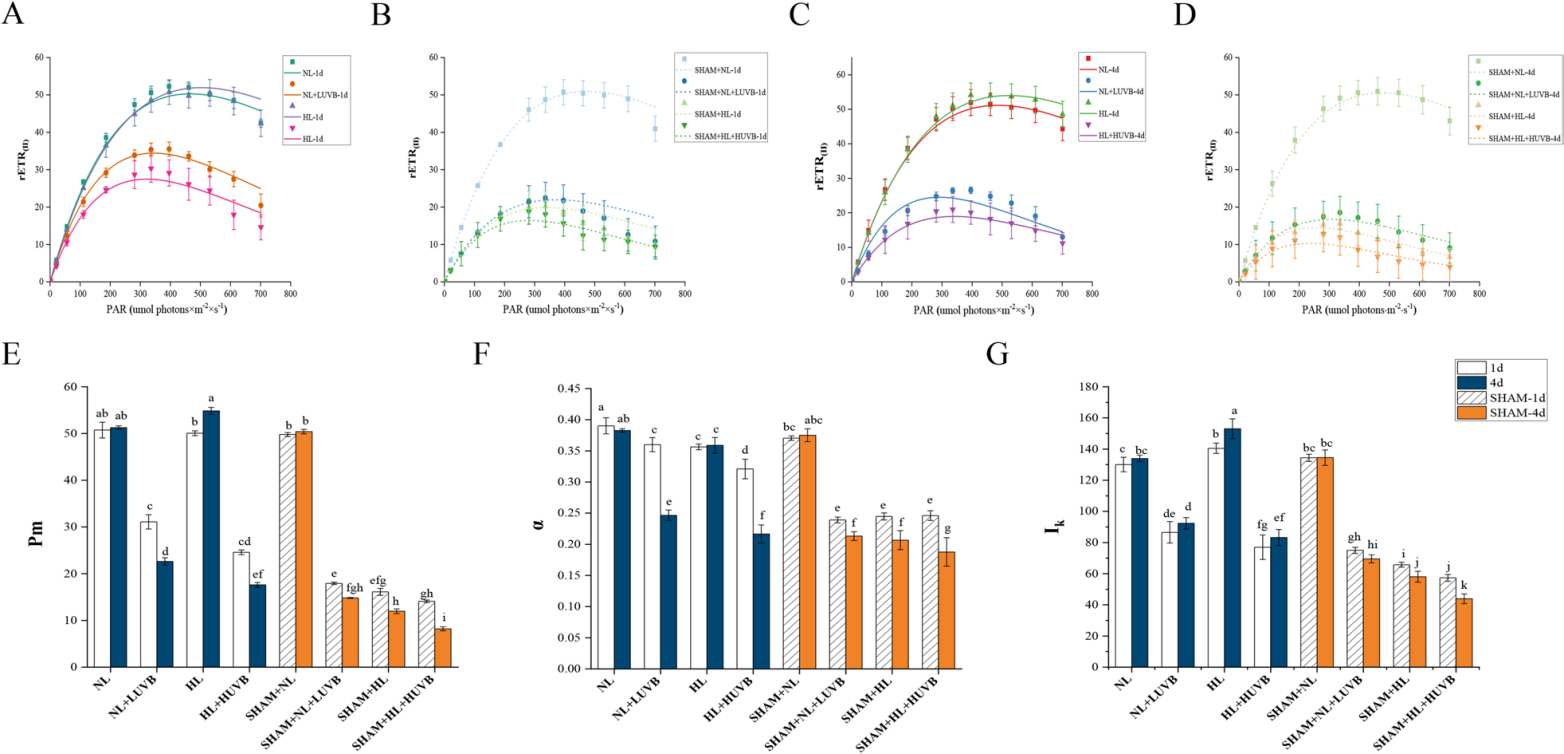
Effects of ultraviolet-B (UVB) and photosynthetically active radiation (PAR) on the relative electron transport rate (rETR) of rapid light response curves (RLCs) and the parameters of the RLCs in *Ulva prolifera* on the 1st day and the 4th day. **A** The relative electron transfer rate (rETR) of the RLCs on the 1st day in the SHAM-free group; **B** the rETR of the RLCs on the 1st day in the SHAM group; **C** the rETR of the RLCs on the 4th day in the SHAM-free group; **D** The rETR of the RLCs on the 4th day in the SHAM group; **E** maximum relative electron transfer rate (Pm); **F** photosynthetic rate in the light-limited region of the RLC (α); **G** minimum saturated irradiance (I_k_). SHAM-1d and SHAM-4d show the changes of the SHAM group on the 1st day and the 4.^th^ day, respectively. All data are the mean values (± SD) from three biological replicates. Groups with different lowercase letters are significantly different (*P* < 0.05)

As shown in Fig. 4, the results of the rapid light curves (RLCs) revealed the mean relative electron transfer rate (rETR) on the 1st day and 4th day. As the irradiation regime was intensified, the values of the rETR decreased significantly. The inhibition of the rETR was more pronounced with the presence of SHAM. As can be seen in Fig. 4, the maximum relative electron transfer rate (Pm), the photosynthetic rate in the light-limited area of the RLC (α), and the minimum saturated irradiance (I_k)_ all showed significant differences (*P* < 0.05) with the increasing irradiation intensity and irradiation time, and a further significantly decreasing trend was seen with the application of SHAM.

### ***Detection of nicotinamide adenine dinucleotide phosphate hydrogen***

**Fig. 5:**
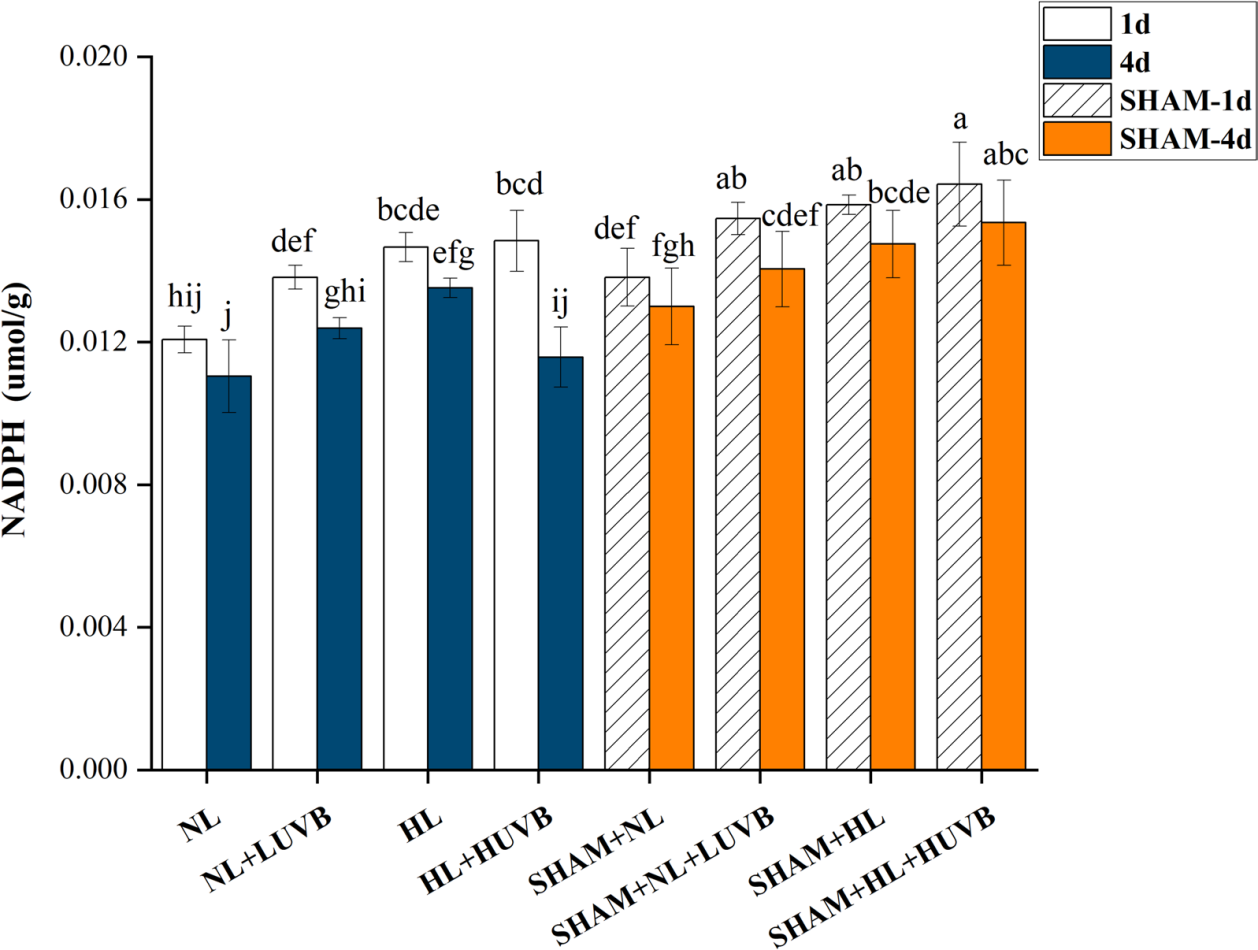
Effects of ultraviolet-B (UVB) and photosynthetically active radiation (PAR) on nicotinamide adenine dinucleotide phosphate hydrogen (NADPH) content in *Ulva prolifera* on the 1st day and the 4th day. SHAM-1d and SHAM-4d show the changes of the SHAM group on the 1st day and the 4.^th^ day, respectively. All data are the mean values (± SD) from three biological replicates. Groups with different lowercase letters are significantly different (*P* < 0.05)

As illustrated in Fig. 5, compared with the NL radiation, the contents of nicotinamide adenine dinucleotide phosphate hydrogen (NADPH) increased by 14.4%, 20.9%, and 22.9% under NL + LUVB, HL, and HL + HUVB radiation on day 1, respectively. The NADPH content increased by 12.19% and 19.13% under NL + LUVB and HL radiation on day 4, respectively. Furthermore, the content of NADPH on day 4 was significantly lower than that on day 1. The content of NADPH increased significantly in the presence of SHAM compared with the thalli without SHAM.

### Changes in UVB and PAR caused ROS production

**Fig. 6:**
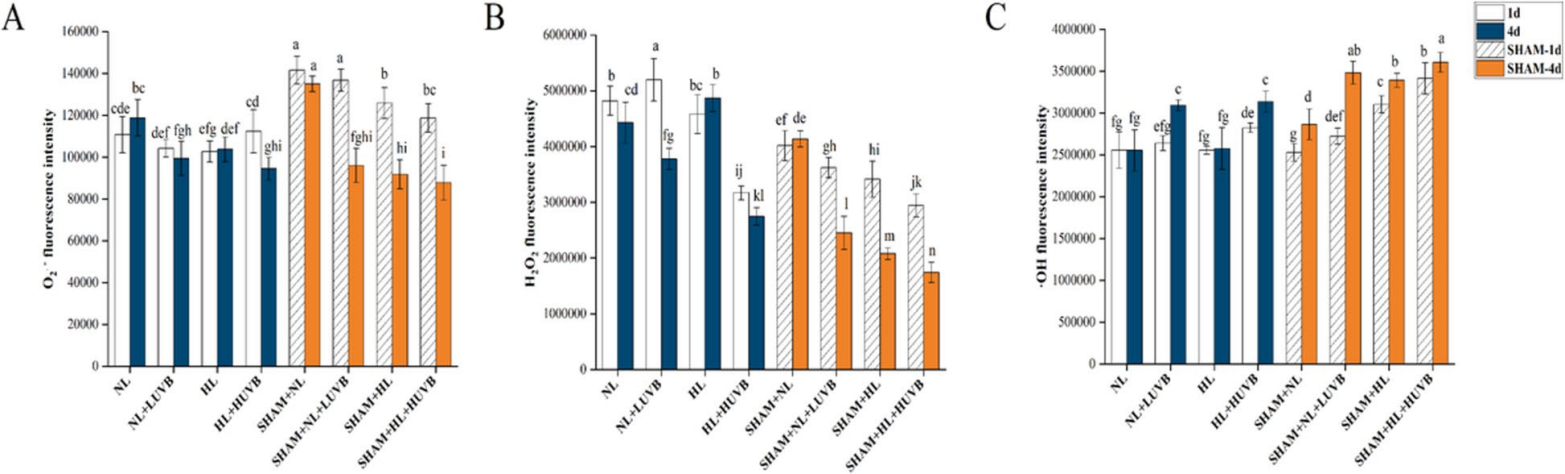
Effects of ultraviolet-B (UVB) and photosynthetically active radiation (PAR) on the reactive oxygen species (ROS) levels in *Ulva prolifera* on the 1st day and the 4th day. SHAM-1d and SHAM-4d show the changes of the SHAM group on the 1st day and the 4.^th^ day, respectively. **A** ${{\text{O}}}_{2}^{\bullet -}$ levels; **B** H_2_O_2_ levels; **C** $\bullet {\text{OH}}$ levels. All data are the mean values (± SD) from three biological replicates. Groups with different lowercase letters are significantly different (*P* < 0.05)

The fluorescence intensity of the superoxide anion (${{\text{O}}}_{2}^{\bullet -}$) decreased by 16.4% and 20.5% under UVB radiation compared with the thalli under NL radiation on the 4th day, respectively (Fig. 6-A). The hydrogen peroxide (H_2_O_2_) fluorescence intensity of the thalli under NL + LUVB radiation was 1.08 times higher than that of the thalli under NL radiation, while it was reduced by 34.2% on day 1 under HL + HUVB radiation. The fluorescence intensity of the thalli under UVB radiation decreased significantly on the 4th day (Fig. 6-B). Only the thalli showed a significant increase in the fluorescence intensity of the hydroxyl radical ($\bullet {\text{OH}}$) on day 1 under HL + HUVB radiation. Under NL + LUVB and HL + HUVB radiation, the fluorescence intensity of the thalli was elevated by 21% and 22.8% on the 4th day, respectively (Fig. 6-C).

The fluorescence intensity of the ${{\text{O}}}_{2}^{\bullet -}$ in each treatment group with SHAM was higher than that in the treatment groups without SHAM on day 1 (Fig. 6-A). The ${{\text{O}}}_{2}^{\bullet -}$ fluorescence intensity of the SHAM + NL radiation was significantly higher than that of the NL radiation both on the 1st day and the 4th day. The fluorescence intensity of H_2_O_2_ showed a decreasing trend with the enhancement of the UVB intensity and PAR intensity on the 1st day and the 4th day (Fig. 6-B). The fluorescence intensity of $\bullet {\text{OH}}$ under the SHAM + HL and SHAM + HL + HUVB radiation increased by 17.6% and 17.3% compared to those of the thalli under HL and HL + HUVB on day 1, respectively. On the 4th day, the thalli in the SHAM + NL, SHAM + NL + LUVB, and SHAM + HL + HUVB groups increased by 10.8%, 11.1%, and 13% compared with the thalli without SHAM, respectively (Fig. 6-C).

### Changes in the antioxidant enzyme

**Fig. 7:**
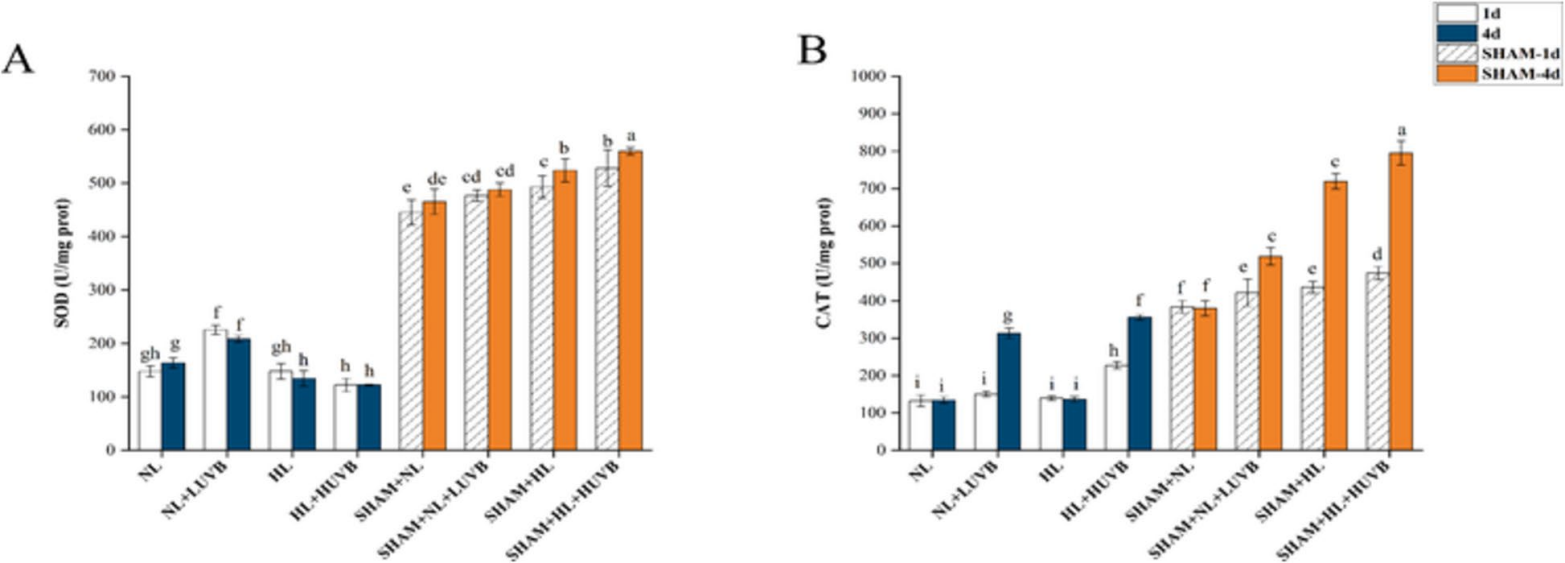
Effects of ultraviolet-B (UVB) and photosynthetically active radiation (PAR) on superoxide dismutase (SOD) and catalase (CAT) activity in *Ulva prolifera* on the 1st day and the 4th day. SHAM-1d and SHAM-4d show the changes of the SHAM group on the 1st day and the 4.^th^ day, respectively. **A** SOD activity; **B** CAT activity. All data are the mean values (± SD) from three biological replicates. Groups with different lowercase letters are significantly different (*P* < 0.05)

As shown in Fig. 7-A, the superoxide dismutase (SOD) activity of the thalli under NL + LUVB was 1.53 and 1.27 times higher on the 1st day and the 4th day in the absence of SHAM compared with the NL radiation, respectively. The HL + HUVB radiation was 25% lower than the NL radiation on the 4th day. The SOD activity of the thalli with SHAM increased significantly compared to that without the inhibitor.

In the absence of SHAM, the CAT activity under the HL + HUVB radiation was 70.7% higher than that under NL radiation on day 1. On the 4th day, the CAT activity under NL + LUVB and HL + HUVB radiation was 1.35 and 1.7 times higher than that under NL, respectively. The CAT activity of the thalli increased significantly with the application of SHAM. The upward trend was particularly significant on the 4th day (Fig. 7-B).

### Effect of the changes in the UVB and PAR on the Rubisco and GO activity

**Fig. 8:**
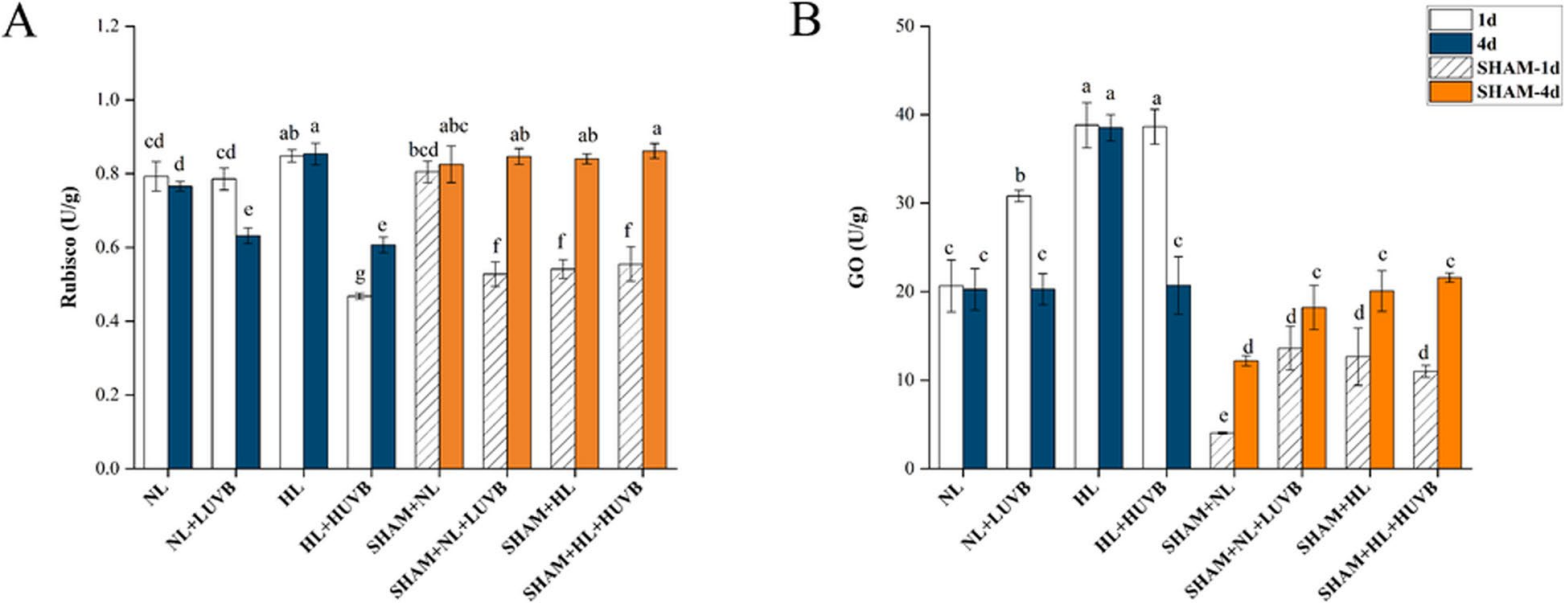
Effects of ultraviolet-B (UVB) and photosynthetically active radiation (PAR) on Rubisco and glycolate oxidase (GO) activity in *Ulva prolifera* on the 1st day and the 4th day. SHAM-1d and SHAM-4d show the changes of the SHAM group on the 1st day and the 4.^th^ day, respectively. **A** Rubisco activity; **B** GO activity. All data are the mean values (± SD) from three biological replicates. Groups with different lowercase letters are significantly different (*P* < 0.05)

As shown in Fig. 8-A, the ribulose-1,5-diphosphate carboxylase (Rubisco) activity in the HL radiation group significantly increased by 6.8%, while the Rubisco activity under HL + HUVB radiation was significantly reduced by 40.9% on the 1st day. The Rubisco activity under NL + LUVB radiation decreased by 17.6%, and that under HL + HUVB radiation decreased by 21.2% compared with the thalli under NL radiation on the 4th day. The Rubisco activity of the thalli under HL radiation was significantly elevated on both the 1st day and the 4th day. After the application of SHAM, the Rubisco activity of the SHAM + NL group was significantly higher than that of the NL group. The Rubisco activities of the thalli under UVB or HL groups decreased significantly compared with the NL group with SHAM on the 1st day. However, on the 4th day, the Rubisco activities of the SHAM + NL + LUVB, SHAM + HL, and SHAM + HL + HUVB groups were significantly elevated.

As shown in Fig. 8-B, the glycolate oxidase (GO) activities in the NL + LUVB, HL, and HL + HUVB groups increased by 49.2%, 88%, and 87.03% compared with the thalli under NL radiation on the 1st day, respectively. On the 4th day, the GO activity of the HL group significantly increased by 86.6%. However, the GO activity decreased significantly with the presence of SHAM on the 1st day.

### Results of RNA sequencing in U. prolifera under changes of UVB

**Fig. 9:**
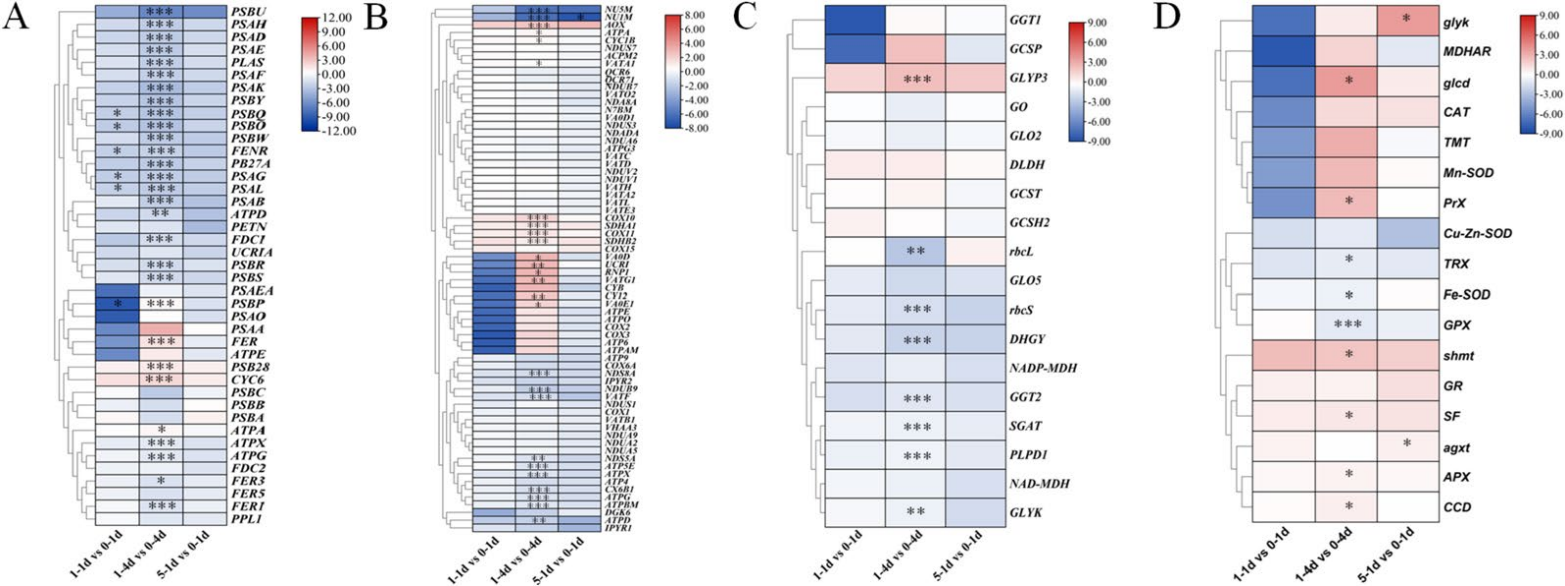
Agglomerative hierarchical clustering of **A** 41 photosynthesis-related differentially expressed genes (DEGs). **B** Sixty-eight respiration-related DEGs; **C** 18 photorespiration-related DEGs; **D** 17 antioxidant system-related DEGs in Ulva prolifera after the 1st day of 1 W/m^2^ ultraviolet-B (UVB) (1-1d), after the 4th day 1 W/m^2^ UVB (1-4d), after the 1st day of 5 W/m^2^ UVB (5-1d) compared with the control. In the figure, 0-1d indicates after the 1st day of 72 μmol m^−2^ s^−1^ photosynthetically active radiation (PAR); 0-4d indicates after the 4th day of 72 μmol m^−2^ s.^−1^ PAR. The color range from blue to red indicates the degree of enrichment from low to high, respectively. Statistical significance is indicated by * (*P* < 0.05), **(*P* < 0.01), and ****(P < 0.001)

The differentially expressed genes (DEGs) of the thalli treated with UVB radiation are summarized in Fig. 9, including DEGs in the “photosynthesis”, “respiration”, “photorespiration”, and “antioxidant system”. A total of six DEGs related to photosynthesis decreased significantly under NL + LUVB radiation on day 1. Five DEGs related to photosynthesis increased significantly, and 24 DEGs related to photosynthesis decreased significantly under NL + LUVB radiation on day 4. Fourteen DEGs related to respiration increased significantly, and 12 DEGs related to respiration decreased significantly under NL + LUVB radiation on day 4. Only the DEG of *NU1M* decreased significantly under HL + HUVB radiation on day 1. The DEG of *GLYP3* increased significantly, and seven DEGs decreased significantly under NL + LUVB radiation on day 4. A total of six DEGs increased significantly, and three DEGs decreased significantly under NL + LUVB radiation on day 4. The DEGs of *glyk* and *agxt* increased significantly under HL + HUVB radiation on day 1.

### Principal component analysis

**Fig. 10:**
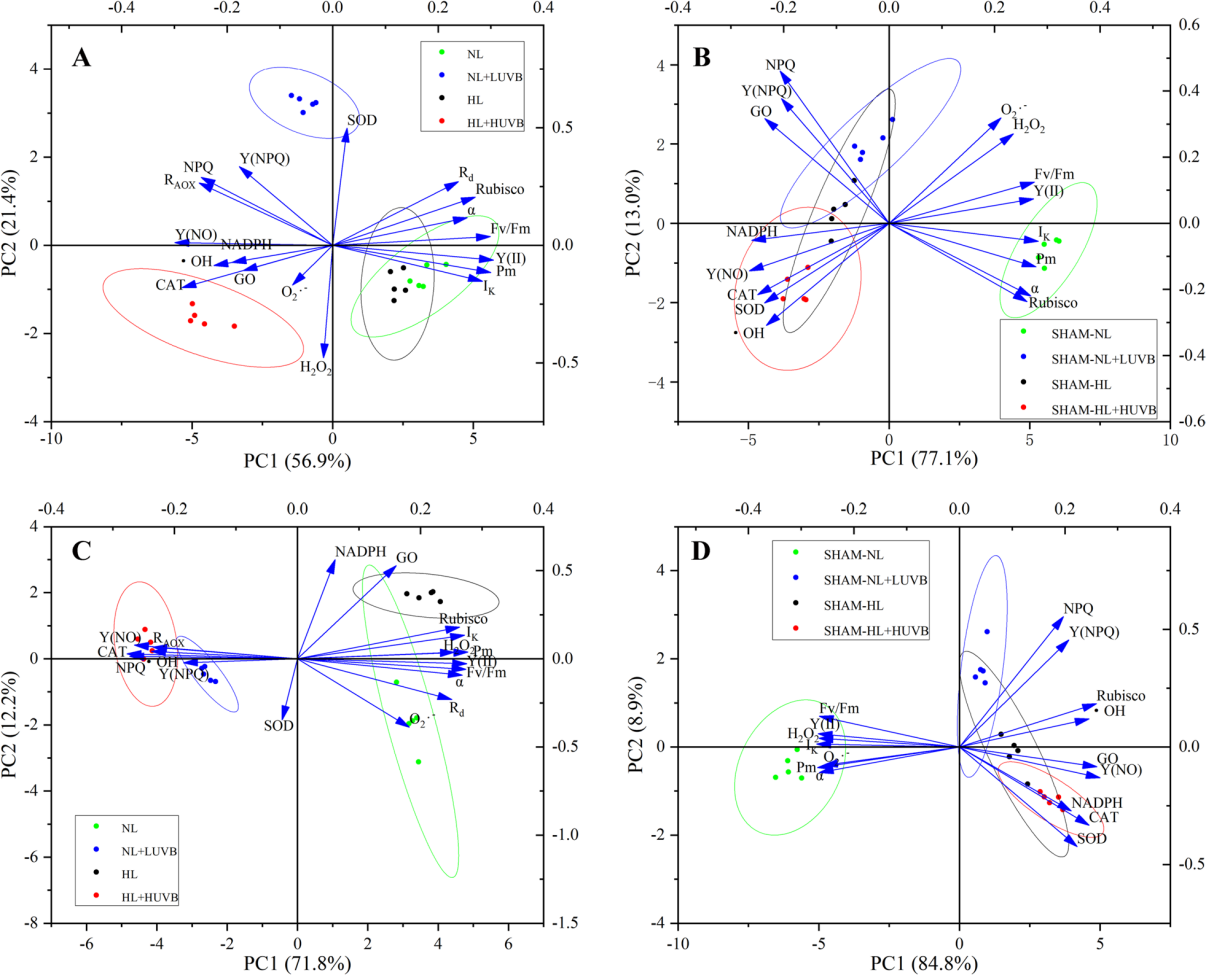
Principal component analysis in the test index in the different groups of *Ulva prolifera*. **A** Principal component analysis of the SHAM-free group on the 1st day; **B** principal component analysis of the SHAM group on the 1st day; **C** principal component analysis of the SHAM-free group on the 4th day; **D** principal component analysis of the SHAM group on the 4th day

We analyzed the relationship between the radiation intensity and the key physiological processes in the thalli using principal component analysis (PCA) (Fig. 10). SOD was the main related physiological process under NL + LUVB radiation, and CAT and GO were the main related physiological processes under HL + HUVB radiation on day 1 without SHAM. GO, Y(NPQ) and NPQ were the main related physiological processes under NL + LUVB radiation, and Y(NO), CAT, and SOD were the main related physiological processes under HL + HUVB radiation on day 1 with the application of SHAM. Y(NPQ) was the main related physiological process under NL + LUVB radiation, and R_AOX_, Y(NO), CAT and NPQ were the main related physiological processes under HL + HUVB radiation on day 4 without SHAM. Y(NPQ) and NPQ were the main related physiological process under NL + LUVB radiation, and CAT, Y(NO), and GO were the main related physiological processes under HL + HUVB radiation on day 4 with the application of SHAM.

## Discussion

*U. prolifera*, as a type of macroalga in the intertidal zone, is intermittently exposed to changes of solar radiation [13, 39]. PAR and UVB, as the important components of solar radiation, displayed different effects on the thalli. As a bypass of respiratory electron transfer, AOX plays important roles in ROS homeostasis and photosynthesis optimization under stresses in higher plants, while little research has focused on the role of AOX during the adaptation of macroalgae to PAR and UVB. In this research, we detected the respiratory rate of AOX, chlorophyll fluorescence, ROS contents, Rubisco activity, GO activity, and antioxidant enzyme activity to explore the response strategy of thalli under changes in PAR and UVB. This research provides a theoretical basis for understanding the adaptive mechanism of plants to adversity and accurately assessing the environmental impact of solar radiation.

### ***Relationship among AOX, photosynthesis and photorespiration***

Macroalgae showed strong protection potential under strong light irradiation, especially under enhanced UVB conditions. NPQ plays important roles in acclimating to the environment under conditions of UVB radiation in macroalgae [16, 18, 40]. AOX was found to be sensitive to UVB stress and participated in the response process of thalli to UVB [33, 41, 42]. UVB, as a common type of abiotic stress, has been widely studied and found to have a significant inhibition on photosynthetic components in macroalgae [16, 18, 43]. It has also been confirmed that AOX plays an important role in maintaining the normal operation of photosynthesis when plants are under abiotic stresses [22, 26, 44]. In macroalgae, AOX plays an important role during the stress resistance process, and AOX is essential in maintaining the redox homeostasis of algal chloroplast development during greening [30, 37]. Compared with the control group (NL), the AOX activity of thalli increased significantly under PAR + UVB radiation, while there was no significant change in the thalli under HL radiation.

PS II is the key site of UVB radiation damage. The UVB radiation enhancement can directly degrade the reaction center protein of PS II, making its structure irreversibly damaged and inactivated. In addition, electron transfer is notably blocked in macroalgae [18, 40, 45–48]. This study found that the photosynthetic activity of the thalli was significantly reduced under UVB radiation. UVB radiation significantly reduced the *Fv/Fm* and Y(II) of *U. prolifera*, and the inhibitory effect of HL + HUVB radiation was significantly greater than that of NL and NL + LUVB radiation. With an increase in UVB radiation, the maximum relative electron transfer rate (rETRmax), α, and I_k_ decreased significantly, indicating that the electron transfer ability of *U. prolifera* decreased significantly under UVB radiation. Additionally, the existence of SHAM led to a further reduction in the photosynthetic activity and further increased the degree of light damage.

Rubisco plays a key role during the process of photosynthetic carbon assimilation [49]. Rubisco activity significantly decreases under high levels of UVB radiation in the macroalgae *Ulva lactuca* and *Monostroma arcticum* [50, 51]. Similar results were observed in our present study, in which the Rubisco activity decreased significantly under UVB radiation in *U. prolifera*. Furthermore, the Rubisco activity of each treatment group with SHAM was higher than that of the NL group on day 4 compared with those with SHAM, which indicated the complementarity between AOX and Rubisco in *U. prolifera*.

GO is the key enzyme of photorespiration. The activity of GO increased on day 1, and the result of PCA analysis confirmed that there was a strong correlation between GO activity and NADPH homeostasis. NADPH homeostasis is an important basis for maintaining the operation of photosynthesis in macroalgae [35]. With an increase in the radiation intensity, the content of NADPH increased, especially after the applied SHAM. This was consistent with the change in the NADPH content in *Capsicum annuum* under drought stress [52]. In addition, it is generally believed that the optimal mechanism of plant photosynthesis by an alternative pathway is to dissipate excess reduced equivalents in the chloroplasts through the malate/oxaloacetate (Mal/OAA) shuttle [53, 54]. However, some studies have found that an AOX respiratory pathway can also maintain or increase the operation of photorespiration, reduce the over-reduction of photosynthetic electron transport chain, and detoxify glycolate, thus possibly playing a protective role against photodamage of PS II in macroalgae [35]. GO activity can be used to indicate the photorespiration flux [55, 56]. Our previous research has confirmed that GO activity is positively correlated with other key enzymes, namely serine: glyoxylate aminotransferase (SGAT) and glycerate kinase (GLYK), in the photorespiration pathway when *U. prolifera* is exposed to short-term and low-intensity UVB radiation [43]. In addition, rice plants induced by antisense GO showed an obvious photorespiration defect phenotype [57], and in related research on *Jatropha curcas* plants under severe drought stress, GO activity was used to further verify the occurrence of photorespiration [58]. In this study, it was found that the GO activity decreased significantly when AOX was inhibited, and this suggested that the inhibition of AOX hindered the normal operation of GO [35, 59].

In this study, the activity of GO significantly increased under HL conditions. GO, as a key enzyme of photorespiration, plays an important role in maintaining the reduction equivalent of the thalli [35, 56]. The important role of photorespiration in the adaptation of *U. prolifera* to stresses has been demonstrated in our previous studies [43]. In the present study, under HL conditions, AOX regulated the redox equivalent within the photosynthetic system by modulating the photorespiration process with GO as the key enzyme, so as to avoid an excessive reduction equivalent and optimize the photosynthetic activity of the thalli. Based on PCA analysis, the AOX-mediated optimization of photosynthesis worked synergistically with the NPQ-dependent photoprotection process to achieve a balanced photoprotection mechanism in the algae. In this study, the I_k_ of algae under HL conditions was significantly up-regulated. As the parameter of minimum saturating irradiance, I_k_ can well characterize the tolerance of algae to HL. This indicator can further reflect the strong ability of the thalli to resist high light stress at this time [60, 61]. Therefore, at this time, the NPQ of the thalli did not show a significant increase compared to those under NL conditions. The absence of AOX led to a loss of GO activity, resulting in an imbalance in the reduction equivalent and a weakened ability of the thalli to withstand stress [35]. The photosynthetic activity of the thalli significantly decreased in the absence of AOX, despite relying in a greater NPQ development. The phenomenon of the rapid loss of photosynthetic activity and irreversible damage may occur if the short-term protective pathways are insufficient to protect PS II [62]. Similar results were also observed in previous researches [16, 43, 63]. This inference was further supported by the results of Y(NPQ) and Y(NO). Compared to the SHAM + NL + LUVB treatment, significant decrease in Y(NPQ) and significant increase in Y(NO) could be observed in the thalli under the SHAM + HL + HUVB treatment. This indicated that the level of photodamage on the thalli has exceeded their protective capacity. Interestingly, previous studies indicated that the NPQ of *U. prolifera* significantly increased under stressors, but its NPQ value was relatively low compared to other species [63–66]. Further investigation about its mechanism is necessary in the future.

### Relationship between AOX and ROS homeostasis

The changing trend of ${{\text{O}}}_{2}^{\bullet -}$ in the NL + LUVB group showed no significant change compared with the thalli under NL radiation. A similar result was reported in rice under low intensity of UVB [67]. In addition, the response of *U. prolifera* under a low intensity of UVB is different [18]. A reasonable explanation was that ${{\text{O}}}_{2}^{\bullet -}$ was transitorily up-regulated during the early stage because of the relatively stable value of α, and this could ensure that photosynthesis continuously produced ${{\text{O}}}_{2}^{\bullet -}$. SOD plays an important role in the consumption of ${{\text{O}}}_{2}^{\bullet -}$ in macroalgae, and the significant increase in SOD at this time would further consume ${{\text{O}}}_{2}^{\bullet -}$ [18, 68]. However, this may have been related to the increased activity of AOX at this time. On the 4th day, the content of ${{\text{O}}}_{2}^{\bullet -}$ in the thalli significantly decreased. However, under the HL + HUVB conditions, the SOD activity in the thalli was relatively low. This may have been due to the reaction of ${\mathrm{ O}}_{2}^{\bullet -}$ with H_2_O_2_, causing a significant decrease in the H_2_O_2_ content and a simultaneous significant increase in the ·OH content. At this time, the enhanced CAT activity intensified the downward trend of H_2_O_2_. It had been proven that AOX can directly inhibit the production of ROS and also indirectly affect the scavenging of ROS by increasing the intensity of the ROS scavenging network [18]. The significant increase in the ${{\text{O}}}_{2}^{\bullet -}$ content after the application of SHAM supported this point. The time change trend of the HL + HUVB group was consistent with *U. prolifera* [18], but it was different on the 1st day. This may have been related to the enhancement of PAR in this experiment. The research showed that properly increasing the strength of PAR was helpful to reduce the damage caused by UVB [69]. After the applied SHAM, the ${{\text{O}}}_{2}^{\bullet -}$ content significantly increased, and this induced an increase in the SOD activity on the 1st day. These results were consistent with those of tobacco treated with antimycin A [70], but the increase in SOD could not make up for the increase $\mathrm{in the}{\mathrm{ O}}_{2}^{\bullet -}$ content caused by AOX inhibition. Hence, the ${{\text{O}}}_{2}^{\bullet -}$ content significantly increased, similar to the reaction of tomatoes at low temperatures [27].${{\text{O}}}_{2}^{\bullet -}$ was negatively correlated with SOD and $\bullet {\text{OH}}$ on day 4, indicating that a decrease in ${{\text{O}}}_{2}^{\bullet -}$ was related to an increase in SOD and the formation of $\bullet {\text{OH}}$.

With ${{\text{O}}}_{2}^{\bullet -}$ as the substrate, H_2_O_2_ can be generated under the catalysis of SOD, and H_2_O_2_ can be further decomposed under the catalysis of CAT [71]. Therefore, an increase in the H_2_O_2_ content in the NL + LUVB group was related to the increase in the SOD activity and no obvious change in the CAT activity. This was consistent with an increase in the H_2_O_2_ concentration when tobacco was exposed to a 170 − 180% environmental intensity of UVB [72]. The decrease in the HL + HUVB group was related to an increase in CAT. The PCA analysis indicated that there was a positive correlation between R_AOX_ and CAT. After the SHAM was applied, the total amount of H_2_O_2_ decreased, which was consistent with the results of tobacco and *Arabidopsis* [70, 73, 74]. This confirmed the overcompensation of the H_2_O_2_ scavenging system in cells and the production of hydroxyl radicals as substrates that was demonstrated by the changes in the CAT activity and $\bullet {\text{OH}}$ in this study.

$\bullet {\text{OH}}$ is produced from ${{\text{O}}}_{2}^{\bullet -}$ and H_2_O_2_ as the substrate via the Harber-Weiss reaction [75], which is the primary substance that causes oxidative damage. With an increase in the irradiation intensity, the $\bullet {\text{OH}}$ in *U. prolifera* increased and the concentration of $\bullet {\text{OH}}$ further increased after SHAM was applied. The PCA analysis showed that AOX was positively correlated with $\bullet {\text{OH}}$ under high-intensity UVB radiation. This indicated that AOX was related to the protection of thalli after oxidative damage. This protective process is not sufficient to repair the oxidative damage of macroalgae [76].

The H_2_O_2_ content in the thalli increased significantly under NL + LUVB, and this was related to the up-regulation of SOD activity. The ${{\text{O}}}_{2}^{\bullet -}$ produced by photosynthesis provided the substrate for H_2_O_2_ production. PAR + UVB induced a significant increase in the $\bullet {\text{OH}}$ content, and this was related to the oxidative damage of the macroalgae. The CAT activity increased significantly under PAR + UVB, while it could not completely remove the excess $\bullet {\text{OH}}$ in the macroalgae. The synergism of AOX with SOD and CAT played an important role during the process of ROS homeostasis under PAR + UVB.

## Conclusions

**Fig. 11:**
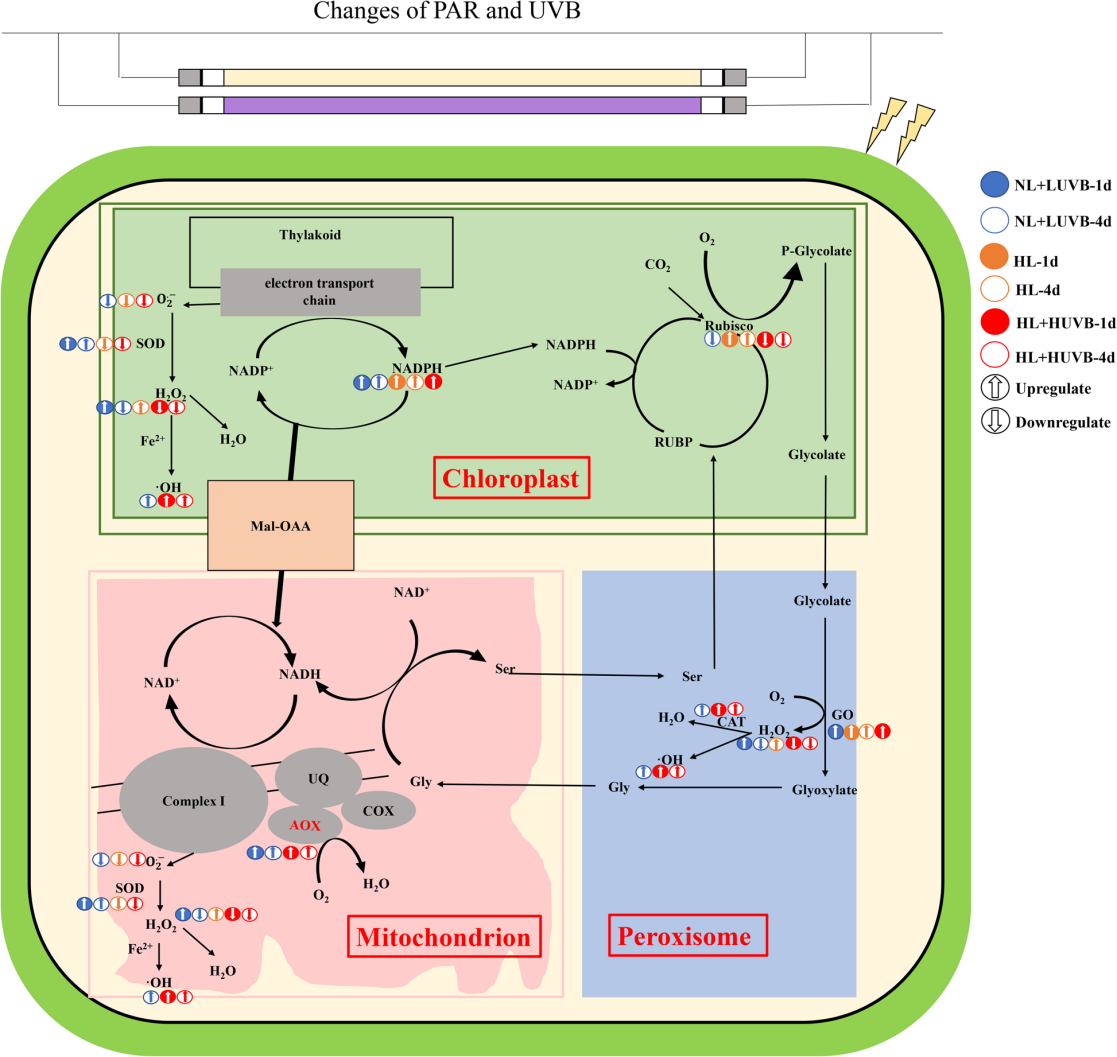
Physiological process of *Ulva prolifera* under changes in photosynthetically active radiation (PAR) and ultraviolet-B (UVB)

In summary, UVB is the main component of solar radiation that impacts the typical intertidal green macroalgae *U. prolifera*. AOX is an important response process in *U. prolifera* under changes in PAR and UVB (Fig. 11). AOX plays an important role during the process of photosynthesis optimization of *U. prolifera* due to a synergistic effect with NPQ under UVB radiation. AOX and GO work together to accomplish NADPH homeostasis to achieve photosynthesis optimization under changes in PAR + UVB. The synergism of AOX with SOD and CAT is important during the process of ROS homeostasis under PAR + UVB. This study provides further insights into the response of intertidal macroalgae to solar light changes.

## Materials and methods

### Experimental design

The experiment was undertaken on free-floating thalli of *U. prolifera* that were collected from coastal Qingdao during the bloom period [77]. The thalli were cleaned gently with a brush, and sterile seawater was used to remove the attached sediment, small grazers, and epiphytes. The thalli were then cultured in sterile seawater and enriched with f/2 medium. The culture temperature was set at 20°C, and the light intensity was set at 72 µmol m^−2^ s^−1^ with a 12: 12 h light: dark cycle in a GXZ280C intelligent illumination incubator (Jiangnan, China). To inhibit the growth of diatoms, germanium dioxide (GeO_2_) at a concentration of 0.5 mg/L was used. The media was renewed every two days [18].

### Treatment with inhibitors and UVB

To block the respiration rate of AOX, we used the AOX inhibitor SHAM. In order to understand the inhibition effect of AOX, the activity of AOX was measured with 0.3 mM, 0.6 mM, 1 mM, 2 mM, and 3 mM SHAM (Supplementary Figure S2). Furthermore, the thalli were placed in sterile seawater containing 2 mM SHAM for 3 h to study the internal relationships among AOX, photosynthesis, and ROS homeostasis. The incubated thalli were then rinsed in sterile seawater [78].

UVA can cause both inhibitory and enhancing effects on biomass accumulation, morphology and photosynthesis in plants. However, UVA, which has the longest wavelength compared with UVB, is less efficient than UVB in mediating some biological responses [1]. Although UVB radiation accounts for only a small proportion of sunlight, it can easily affect the growth and reproduction of organisms, physiology, and the redox state, and causes DNA damage in plants [2]. Therefore, in this study, we focused on the response characteristics of *U. prolifera* to UVB. Based on our previous research, the average UVB exposure at Qingdao during the summer was 2.29 W/m^2^, and the low- and high-intensity UVB in this experiment were set at 1 W/m^2^ and 5 W/m^2^, respectively [18]. The low and high intensity of the PAR were set at 100 μmol m^−2^ s^−1^ and 400 μmol m^−2^ s^−1^, respectively, which was referenced to the value of PAR in the Yellow Sea (approximately 100 μmol m^−2^ s^−1^ in late June and 240 μmol m^−2^ s^−1^ in early July) [38, 79]. The experimental cycle was four days, and the photoperiod was 6:00 − 18:00 (PAR), during which the thalli were treated with UVB radiation from 10:00 − 14:00. The rest of the time they were placed in a GXZ-280C incubator (20°C, 72 μmol m^−2^ s^−1^). The UVB radiation system consisted of a UVB lamp (Philips TL 40 W/12RS), which provided a UVB radiation source, that was covered with a cellulose acetate film (0.12 mm) to block UVC radiation. The radiation parameter range of the UVB lamp was 290 nm-315 nm, with a peak wavelength of 311 nm. A lamp (Philips TL-D, 36 W) provided the PAR light source.

**Fig. 12:**
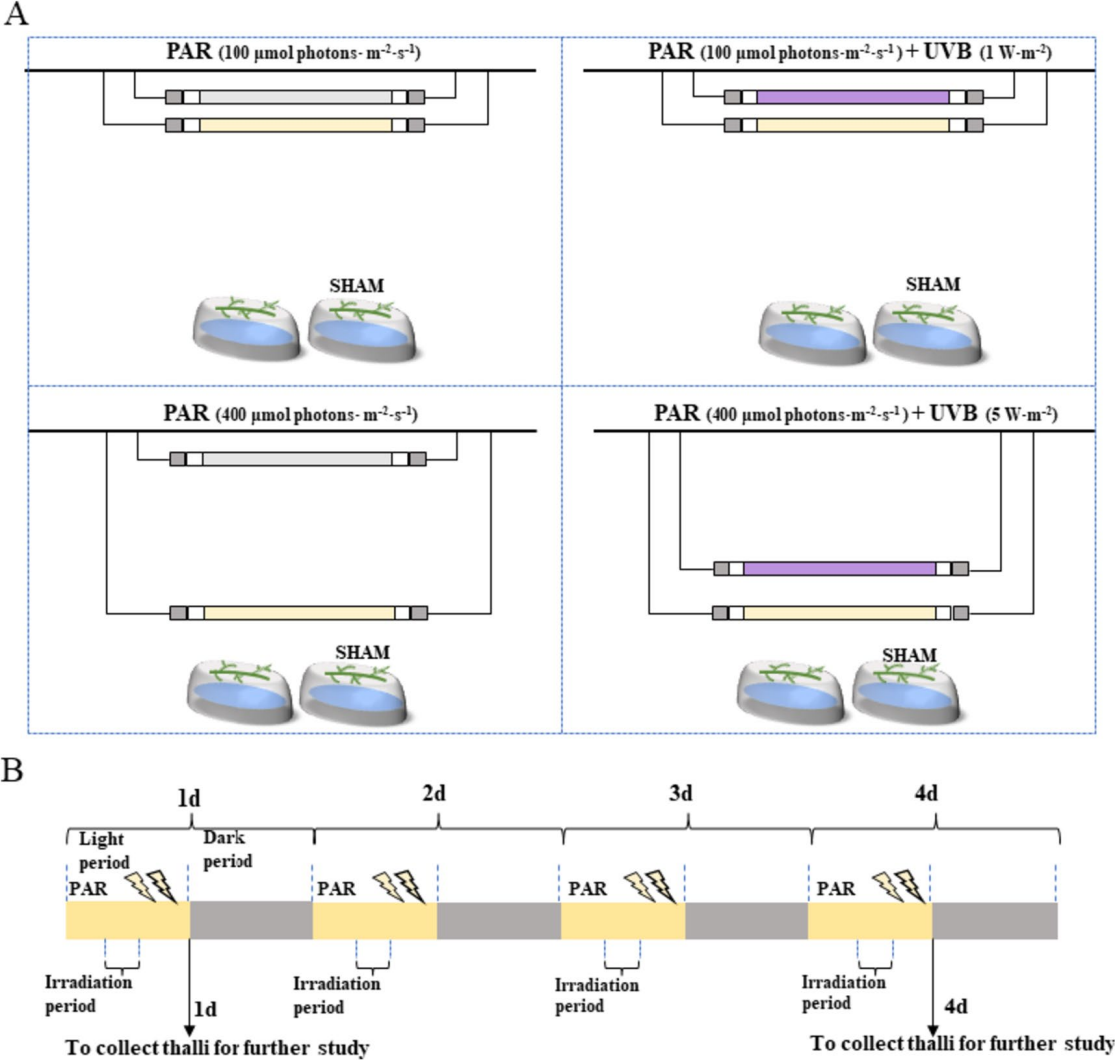
Experimental design of the study

The radiation intensity was changed by changing the distance between the thalli and the lamp. The UVB radiation intensity was measured using the UVB 297 ultraviolet irradiator (Beijing Normal University), and the PAR intensity was determined using the MQ-500 full-spectrum quantum meter (USA). The NL radiation system was PAR:100 μmol m^−2^ s^−1^, the NL + LUVB radiation system was PAR:100 μmol m^−2^ s^−1^ and UVB:1 W/m^2^, the HL radiation system was PAR:400 μmol m^−2^ s^−1^, and the HL + HUVB radiation system was PAR:400 μmol m^−2^ s^−1^ and UVB:5 W/m^2^. The first sampling time was marked as the first day after the first radiation (18:00 on the first day), and the second sampling time was after the fourth radiation (18:00 on the fourth day) and marked as the fourth day (Fig. 12).

### Chlorophyll fluorescence measurements

The chlorophyll fluorescence parameters were measured using imaging-PAM (Heinz Walz, Effeltrich, Germany) to determine the photosynthetic properties of the thalli under the experimental conditions [16]. The thalli were first placed in a 24-well plate containing sterile seawater for 20 min of dark acclimation, and then the induced light curve was measured to determine the *Fv/Fm*, Y(II), Y (NPQ), and Y(NO). The inhibition of PS II due to high PAR or PAR + UVB was calculated as ${{\text{Ihn}}}_{{\text{PAR}}}\left(\mathrm{\%}\right)=\left({Fv/Fm}_{NL}-{Fv/Fm}_{HL}\right) / {FV/Fm}_{NL}\cdot 100$and ${{\text{Ihn}}}_{{\text{PAR}}+{\text{UVB}}}\left(\mathrm{\%}\right)=\left({Fv/Fm}_{NL}-{Fv/Fm}_{PAR+UVB}\right) / {{\text{Fv}}/{\text{Fm}}}_{{\text{NL}}}\cdot 100$, respectively. In addition, the extra UVB-induced inhibition compared with the PAR-only was calculated as ${{\text{Ihn}}}_{{\text{UVB}}}\left(\%\right)=\left({Fv/Fm}_{{\text{PAR}}}-{Fv/Fm}_{{\text{PAR}}+{\text{UVB}}}\right){/Fv/Fm}_{{\text{PAR}}}\cdot 100$ [80]. The samples were exposed to a light intensity gradient (PAR: 0, 1, 21, 56, 111, 186, 281, 336, 396, 461, 531, 611, and 701 μmol photons m^−2^ s^−1^) to measure the RLC using imaging-PAM. The rETR of PSII was obtained by measuring the RLC. Pm, α, and I_k_ were obtained by fitting with Platt's empirical equation:

1 $${\text{P}}={\text{Pm}}\bullet (1-{{\text{e}}}^{-\mathrm{\alpha }\bullet {\text{PAR}}/{\text{Pm}}})\bullet {{\text{e}}}^{-\upbeta \bullet {\text{PAR}}/{\text{Pm}}},$$

2 $${\text{Ik}}={\text{Pm}}/\mathrm{\alpha },$$

where P indicates the rETR; Pm indicates the rETRmax; α is the light energy utilization; and I_k_ reflects the tolerance to intense light [32].

### Measurement of the enzymatic activity

The thalli (0.1 g fresh weight) were ground into a powder form in liquid nitrogen, and 1 ml of 0.05 M phosphate-buffered saline (PBS) (pH = 7.0) was added. This was then centrifuged at 12,000 rpm 4°C for 10 min, and the supernatant was carefully removed to measure the activity of antioxidant enzymes (SOD and CAT) detection). The SOD activity was determined using the cytochrome c reduction method. Inhibition of cytochrome c under experimental conditions reduces the amount of SOD by 50%, and this is defined as one unit of SOD activity [81, 82]. The CAT activity was measured according to previous methods [83, 84]. The extract was mixed with potassium phosphate buffer (1.5 mL at 50 mM, pH = 7.0), 1 mL of deionized water, and 0.3 mL of 0.1 M H_2_O_2_ at 25°C. The CAT activity was measured by recording the absorbance at 240 nm using a spectrophotometer. One unit of CAT activity was defined as the amount of enzyme required to reduce 0.1 absorbance units in the optical density at 240 nm per min.

The thalli (0.1g fresh weight) were rapidly frozen using liquid nitrogen, and 0.9 mL of PBS (pH 7.4) was added to them. Then the homogenization of thalli was completed using an automatic sample rapid grinding apparatus (JXFSTPRP-48L, Shanghai Jing Xin Company). This was followed by centrifugation for 10 min at 10,000 rpm at 4°C. The supernatant was carefully collected for the determination of Rubisco and GO. The kits for detecting the activities of GO (Nanjing Camelot Bioengineering Company, China) and Rubisco (Shanghai Enzyme-linked Biotechnology Company, China) belonged to enzyme-linked immunosorbent assay (ELISA). One hundred microliters of supernatant and biotin-labeled antibody were added to enzyme-labeled plate holes (coated with enzyme antibody in advance) for the reaction, and then washed with PBS. Then, peroxidase-labeled avidin was added for the reaction, washed with PBS, and substrate TMB was added for color development. The color depth was positively correlated with the enzyme activity in the sample. The absorbance was measured at 450 nm using an enzyme-labeled instrument, and the enzyme activity in the sample was calculated based on the standard curve. Sandwich ELISA was used to detect the specific enzyme activity sites in the samples, so as to detect the enzyme activity in the samples. Ten microliters of supernatant and enzyme-labeled reagent were added to the enzyme-labeled coated plate for the reaction. After washing, the substrate was added for color development, and the color development was positively correlated with the enzyme activity in the sample. The absorbance was measured at 450 nm using an enzyme-labeled instrument, and the enzyme activity in the sample was calculated based on the standard curve. The kit used was an ELISA kit, and the Rubisco activity was determined using the double-antibody sandwich method.

### Measurement of the nicotinamide adenine dinucleotide phosphate hydrogen (NADPH) content

The NADPH content was determined strictly following the instructions of the NADPH kit (Beyotime Biotechnology Company, China). The kit used the WST-8 method to detect the content of NADPH in the sample. Approximately 20 mg of the tissue sample was weighed and ground in liquid nitrogen. A total of 400 µL of the extract was then added to homogenize the sample. It was then centrifuged at 12,000 rpm at 4°C for 10 min, and the supernatant was collected to be tested for later use. Two hundred microliters of supernatant were subjected to a 60°C water bath to decompose the NADP^+^ before being heated and centrifuged at 10,000 g for 5 min at room temperature (25℃). A total of 50 µL of supernatant, 100 µL of glucose -6- phosphate dehydrogenase (G6PDH), and 10 µL of chromogenic solution were then mixed and incubated (37℃). The absorbance was measured at 450 nm and the concentration of NADPH in the sample was calculated based on the standard curve.

### Measurement of the ROS content

The thalli (0.1 g fresh weight) were ground into a powder form in liquid nitrogen, and 3 ml of 0.05 M phosphate buffer solution (PBS) (pH = 7.4) was added. This was then centrifuged at 12,000 rpm 4°C for 20 min, and the supernatant was carefully removed to measure the ROS content (${{\text{O}}}_{2}^{\bullet -}$, H_2_O_2_,$\bullet {\text{OH}}$). The supernatant was incubated in 100 µM of hydroethidine (Sigma-Aldrich Co., USA) for 1 h (37°C) without light. The corresponding excitation wavelength was 480 nm, and the emission wavelength was 590 nm to determine the ${{\text{O}}}_{2}^{\bullet -}$ [85]. The supernatant was incubated in 15 µM DCFH-DA (Solarbio Science & Technology Co., Ltd., Beijing) for 45 min (37°C) and protected from light. To determine the H_2_O_2_, the corresponding excitation wavelength was 488 nm and the emission wavelength was 530 nm [86]. The supernatant was incubated in 240 µM of 1,3-cyclohexanedione (CHD) (Sigma-Aldrich Co., USA), 420 mM pH 3.6 NH_4_Ac, and 105 µM of dimethyl sulfoxide (DMSO) under dark conditions for 20 min (95°C). To determine the $\bullet {\text{OH}}$, the corresponding excitation wavelength was 400.5 nm and the emission wavelength was 452.3 nm [87].

### Determination of the respiratory rate

After the thalli were acclimated in the dark for 15 min, the Rd and R_AOX_ were measured using a Chlorolab-3 oxygen electrode (Hansatech, UK) [88]. The experimental temperature was controlled using a water bath circulator at 20°C, and the respiration rate of thalli was obtained based on the slope of the oxygen consumption rate reaching the steady state interval. The Rd was measured under dark conditions without any inhibitor. After the dark respiration measurement, 20 mM SHAM was added to the reaction cup and the respiration rate of thalli was measured under dark conditions (R_SHAM_). The respiration rate of AOX: R_AOX_ = Rd − R_SHAM_. According to the above methods, the Rd and R_AOX_ of thalli under different treatments were measured [35].

### RNA-seq analysis

DEGs associated with photosynthesis, respiration, photorespiration, and the antioxidant system were analyzed under changes in UVB according to Zhao et al. (bioproject number: PRJNA610663, https://www.ncbi.nlm.nih.gov/bioproject/PRJNA610663; biosample number: SAMN14309506, https://www.ncbi.nlm.nih.gov/biosample/SAMN14309506/) [18]. On the basis of this research, the data were further analyzed and organized.

### Statistical analyses

Data are presented as the means of three biological replicates (± SD). The results of the chlorophyll fluorescence, enzyme activity, and ROS content were analyzed using one-way analysis of variance (ANOVA) (SPSS Inc., Chicago, IL, USA) and least significant difference (LSD) tests to analyze the statistical significance among the different groups. Variance homogeneity testing was conducted. The Games-Howell test was performed when the variances of the groups were not homogeneous. The significance level was set at *P* < 0.05. The bar charts, line charts and PCA analysis related to this study were produced using Origin software (OriginLab, Northampton, MA, USA). The DEGs were analyzed using R software with the estimateSizeFactors and nbinomTest DESeq functions. The statistical significance of the DEGs was indicated by * (*P* < 0.05), **(*P* < 0.01), and ***(*P* < 0.001). The cluster analysis of the DEGs was performed using TBtools software (https://github.com/CJ-Chen/TBtools/releases).

### Supplementary Information

**Additional file 1.**

## Data Availability

The analysis data of differentially expressed genes in this study can be obtained from the following website (bioproject number: PRJNA610663, https://www.ncbi.nlm.nih.gov/bioproject/PRJNA610663; biosample number: SAMN14309506, https://www.ncbi.nlm.nih.gov/biosample/SAMN14309506/). Others data generated or analyzed during this study are included in this published article [and its Supplementary information files].
