# Supplementary material for: Acclimation of intertidal macroalgae Ulva prolifera to UVB radiation: the important role of alternative oxidase
Source: BMC Plant Biol. 2024 Feb 28;24:143. doi: 10.1186/s12870-024-04762-w (PMC10900725; doi:10.1186/s12870-024-04762-w)
Supplement: Supplementary file 1 — Additional file 1. [file 12870_2024_4762_MOESM1_ESM.docx]

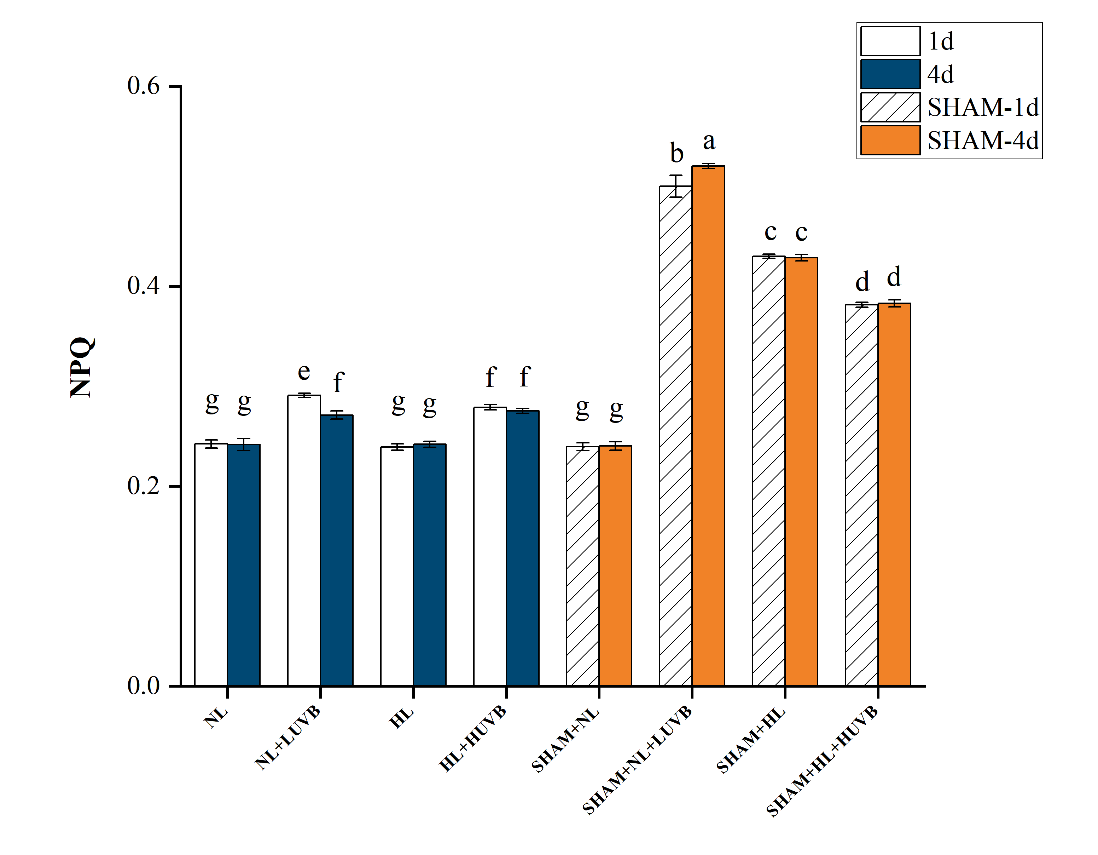


**Fig. S1** Effects of NPQ in *Ulva prolifera* under ultraviolet-B (UVB) and photosynthetically active radiation (PAR) on the 1^st^ day and the 4^th^ day. SHAM-1d and SHAM-4d show the changes of the SHAM group on the 1^st^ day and the 4^th^ day, respectively. All data are the mean values (± SD) from three biological replicates. Groups with different lowercase letters are significantly different (*P* < 0.05).


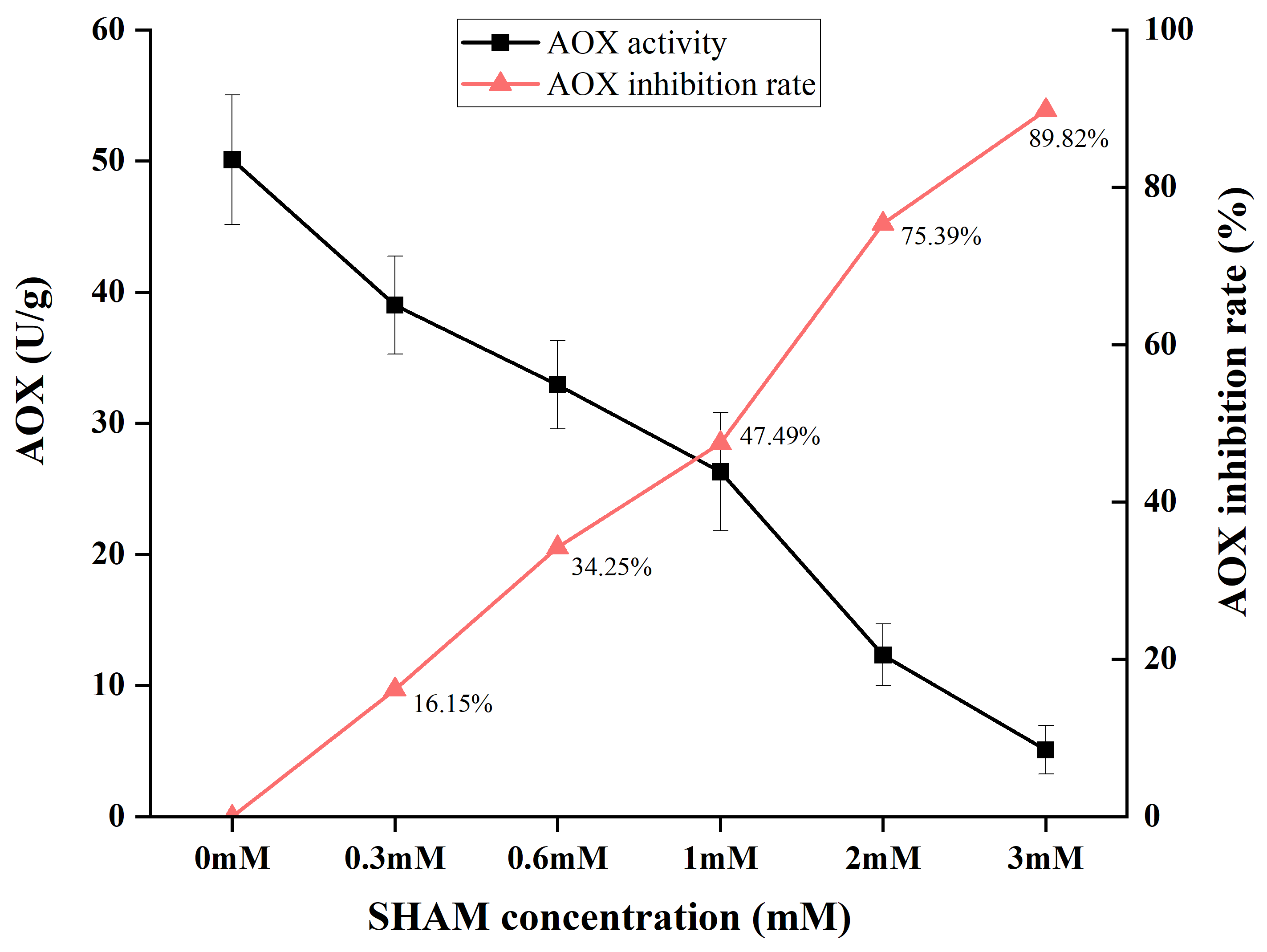


**Fig. S2** Inhibitory rate of different concentrations of SHAM on AOX respiratory pathway. All data are the mean values (± SD) from three biological replicates. Groups with different lowercase letters are significantly different (*P* < 0.05).
